# Patient-specific computational models predict prognosis in B cell lymphoma by quantifying pro-proliferative and anti-apoptotic signatures from genetic sequencing data

**DOI:** 10.1038/s41408-024-01090-y

**Published:** 2024-07-04

**Authors:** Richard Norris, John Jones, Erika Mancini, Timothy Chevassut, Fabio A. Simoes, Chris Pepper, Andrea Pepper, Simon Mitchell

**Affiliations:** 1 Department of Clinical and Experimental Medicine, Brighton and Sussex Medical School, Brighton, UK; 2 School of Life Sciences, University of Sussex, Brighton, UK

**Keywords:** Cancer genetics, B-cell lymphoma, Cell signalling, Apoptosis, Cancer

## Abstract

Genetic heterogeneity and co-occurring driver mutations impact clinical outcomes in blood cancers, but predicting the emergent effect of co-occurring mutations that impact multiple complex and interacting signalling networks is challenging. Here, we used mathematical models to predict the impact of co-occurring mutations on cellular signalling and cell fates in diffuse large B cell lymphoma and multiple myeloma. Simulations predicted adverse impact on clinical prognosis when combinations of mutations induced both anti-apoptotic (AA) and pro-proliferative (PP) signalling. We integrated patient-specific mutational profiles into personalised lymphoma models, and identified patients characterised by simultaneous upregulation of anti-apoptotic and pro-proliferative (AAPP) signalling in all genomic and cell-of-origin classifications (8-25% of patients). In a discovery cohort and two validation cohorts, patients with upregulation of neither, one (AA or PP), or both (AAPP) signalling states had good, intermediate and poor prognosis respectively. Combining AAPP signalling with genetic or clinical prognostic predictors reliably stratified patients into striking prognostic categories. AAPP patients in poor prognosis genetic clusters had 7.8 months median overall survival, while patients lacking both features had 90% overall survival at 120 months in a validation cohort. Personalised computational models enable identification of novel risk-stratified patient subgroups, providing a valuable tool for future risk-adapted clinical trials.

## Introduction

Mutational heterogeneity in haematological malignancies represents a major barrier to reliable prognostication and the development of rationally targeted novel treatment strategies. With the advent of whole exome sequencing (WES) many malignancies, including B cell malignancies such as Diffuse Large B cell Lymphoma (DLBCL) and Multiple Myeloma (MM), have become characterised by interpatient mutational heterogeneity [1, 2]. This heterogeneity contributes to variation in response to current treatments.

The most aggressive haematological malignancies frequently contain genetic aberrations affecting multiple genes, either through co-occurring mutations, e.g. double hit (DH) DLBCL or changes in the copy number of chromosomal regions containing multiple genes, e.g. gain1q MM. DH DLBCL, featuring overexpression of *MYC* and *BCL2* (or *BCL6*), is among the most aggressive lymphoid malignancies, with very poor patient outcomes [3, 4]. However, DH DLBCL represents fewer than 10% of all cases, while many more (30–40%) DLBCL patients relapse following frontline treatment [5]. So, new approaches are clearly needed to prospectively identify poor prognosis patients with the aim of developing more effective treatment strategies for these patients.

Gene expression profiling can split DLBCL into subgroups based on their putative cell of origin (germinal centre -GC, or activated B cell -ABC) [6]. Subsequent studies leveraged genomic sequencing to identify 5 or more prognostically informative patient clusters [7–9]. These genetic groups have been arbitrarily named clusters 1–5 (C1–C5) or with a nomenclature referencing the most mutated signalling pathways (MCD = MYD88 + CD79B); both are broadly consistent across multiple studies (e.g. C5 aligns with MCD) [7–9]. However, clustering patients in this way largely ignores the complexity of the molecular signalling networks that may be impacted by their individual genetic landscapes. For example, a patient with most mutations converging on NF-κB is likely to be assigned to C5 [7], an assignment that ignores the presence or absence of functionally significant co-occurring mutations in other pathways that may impact prognosis.

Computational models of molecular signalling in normal B cells have been used to predict cell proliferation and survival; predictions that have been validated by in vitro laboratory experiments with single-cell resolution [10–13]. Furthermore, incorporating mutations and the impact of mutations on protein abundance/activity, into these models predicts cellular responses in experimental assays [10, 11, 14, 15]. However, it is not known whether these models can predict outcomes at the individual patient scale, nor whether mutational data alone is sufficient to enable in silico simulations to make clinically relevant predictions of prognosis in blood cancers. We hypothesise that contextualising sequencing data within patient-specific, virtual signalling networks may more precisely delineate the consequent cell fate decisions that impact prognosis.

In this study, we used mechanistic computational models to simulate how mutations combine in B cell malignancies. We developed a pipeline to create individual patient simulations using WES and targeted genomic sequencing data, and tested whether these personalised models could generate clinically informative prognostic information.

## Materials and methods

Detailed computational methods, and a lay summary of the methods to generate all computational figures, are provided in the Supplementary Material. Methods are summarised below. Code (as Jupyter notebooks) used to generate and run all computational models, including descriptions of the reactions, parameters and rate laws of each model and code to plot output are available in the Github repository (https://github.com/SiFTW/norrisEtAl).

### Model generation

We employed established computational models of healthy B cells, which enable simulation of proliferation, apoptosis and terminal B cell differentiation in a heterogeneous B cell population [10, 11, 13]. The models consisted of a series of differential equations representing the rate of change of biomolecules (mRNAs, proteins, or protein complexes) over time. Models were converted to Julia to be solved using DifferentialEquations.jl [16, 17].

### Cell cycle and apoptosis modelling

The molecular networks representing apoptosis and the cell cycle were isolated from the comprehensive B cell model [10, 11, 18]. Cell-to-cell variability was simulated by distributing model parameters as described previously [10]. Overexpression of *MYC* and *BCL*2 was simulated by increasing the parameter controlling the transcription rate of the respective mRNA. An equilibrium phase was simulated prior to all time course simulations. For the cell cycle model, the equilibrium phase ended when the value of cell mass reached prior to each cell division was the same (to within 3 decimal places) for 5 consecutive cycles. The final state of the equilibrium phase was used as the initial condition for the time course phase. Cell death was defined as the first time point at which over 10% of poly (ADP-ribose) polymerase (PARP) was cleaved.

### Multi-scale model

The NF-κB component of the established B cell model was updated to include reactions from a more recent and comprehensive model of NF-κB [12]. Apoptosis and cell division were triggered as published previously (cleaved PARP > 2500 triggers apoptosis, CDH1 > 0.1 triggers cell division) [10, 11]. Previous studies introduced two separately simulated daughter cells with each cell division [10, 11, 18], however here this resulted in an unfeasible simulation size in highly proliferative simulations. Cell fates have been shown to be reliably inherited in B cells such that daughter cells will achieve similar fates at similar times [16]. Therefore, at each cell division we replaced the mother cell with a single simulation representing 2^(generation-1)^ daughter cells (2 cells following first division, 4 cells following second division etc).

### Modelling patients

Mutation profiles identified from genomic sequencing and clinical data were downloaded through the cBioPortal [7], or supplements of published studies. We restricted the analysis to genetic changes impacting genes that could be mapped to modelled parameters (https://github.com/SiFTW/norrisEtAl/blob/main/geneList.txt) and leveraged OncoKB to simulate the impact of mutations annotated as ‘likely oncogenic’ [19]. We created 113 patient-specific models using a discovery cohort of DLBCL patients [7], and subsequently validated the findings in 629 patient-specific models using data from 2 validation cohorts of DLBCL patients from recently conducted studies [8, 20]. We assumed half of the normal expression rate of each mRNA could be attributed to one of the two copies of the gene. Therefore, parameters halved for copy number loss and loss-of-function mutations and increased by half (1.5-fold change) for copy number gain and gain-of-function mutations. Copy number alterations of chromosomal regions were modelled by identifying each gene that could be mapped to model parameters within the chromosomal region. Multiple mutations affecting the same parameter were combined multiplicatively. For example a patient with a *MYD88* mutation and a *CD79B* mutation (both increasing NEMO:IKK activity by 1.5-fold individually) was simulated as 2.25-fold increased NEMO:IKK activity. The effect of mutations in genes not modelled directly was assigned to the closest modelled molecular species: e.g. *MCL1* is modelled as overexpression of *BCL2*, and *CARD11* mutations are modelled as increasing NEMO:IKK activity. Patient simulations were performed for 12 simulated hours, on a single-cell with all parameters consistent across all patients other than those impacted by mutations. For simulations of the discovery cohort we simulated 100 cells for each patient and took the mean abundance values for each molecular species. For validation cohorts only one cell was simulated per patient, which was identical in all simulations other than parameters impacted by mutations. The concentration of pro-apoptotic factors (cytoplasmic cytochrome c: cCytoC and second mitochondria-derived activator of caspase: cSmac) and pro-proliferative factor (Cadherin1: Cdh1) at 6 h was used for patient stratification, with patients stratified above or below the mean concentration at that time point.

### Downstream analysis

All model outputs were analysed, and plots generated using the Julia programming language. Scripts relating to each figure can be found in the corresponding folder available in the GitHub repository (https://github.com/SiFTW/norrisEtAl/).

## Results

### Simulating individual mutations recapitulates experimental measurements

**Fig. 1:**
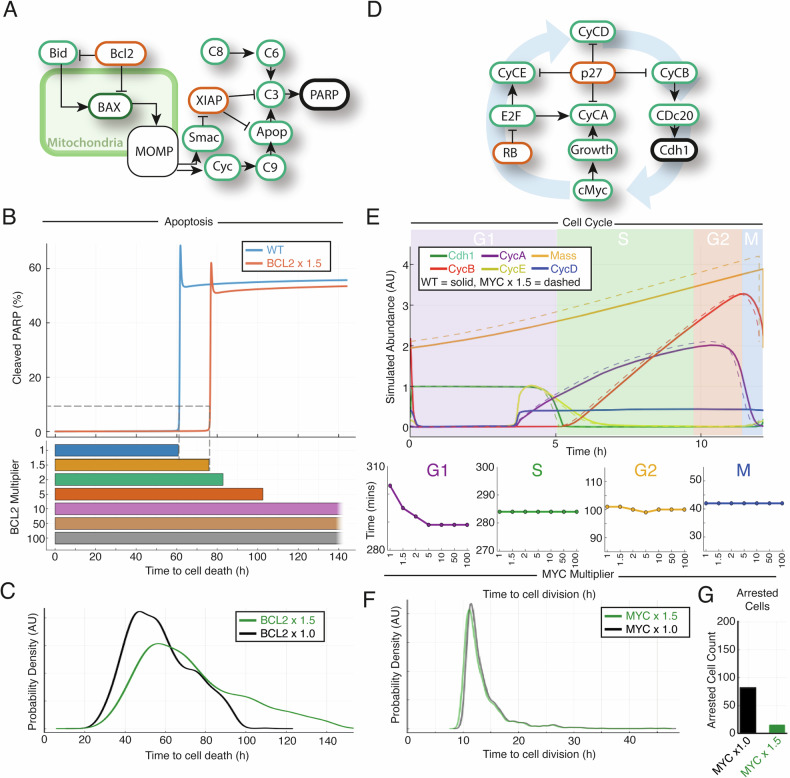
Computational modelling of the cell cycle and apoptosis reveal limited impact of archetypal double hit mutations on their respective molecular networks. **A**–**C** Apoptosis model simulations**. D**–**F** Cell cycle model simulations. **A** Schematic of the apoptosis model leading to cleavage of PARP. Green border: pro-apoptotic regulator, red border: anti-apoptotic regulator, bold black: fate-determining species in the model. **B** (top) The percentage of cleaved PARP over time for two simulations (WT and *BCL2* overexpressed) using the apoptosis model. There is a delay in PARP cleavage in the presence of overexpressed *BCL2*. **B** (bottom) Increasing time to death for cells with increasing *BCL2* expression. **C** Graph showing distribution of time to death in a simulation of 1000 cells, unmutated (black) compared to 1.5-fold *BCL2* upregulation (green). **D** Schematic of the impact of cMyc on the cell cycle model. Green border: pro-apoptotic regulator, red border: anti-apoptotic regulator, bold black: fate-determining species in the model. **E** (top) Output of the abundancies of different cell cycle proteins run to a limit cycle for a WT cell (solid line) and a cell in which *MYC* is overexpressed (dotted line) showing a slight shortening of the cell cycle for some. CycA/D/E = Cyclin A/D/E, Cdh1 = cdc20 homologue 1. **E** (bottom) Time for completion of cell cycle phases for cells with varying *MYC* expression showing the main effect is in G1. **F** Distribution of time to cell division for simulations of heterogeneous populations of 1000 cells. Unmutated (black) compared to 1.5-fold *MYC* upregulation (green) show very little difference. **G** Number of cells, from the simulations in (**F**), for which the cell cycle has arrested in unmutated cells and cells in which *MYC* is upregulated 1.5-fold. Upregulated *MYC* substantially reduces the number of cells in cell cycle arrest.

To determine whether established computational models of B cell molecular signalling could make predictions of patient outcomes we first focused on DH DLBCL. The apoptotic regulatory network was isolated from an established B cell model, and we simulated the impact of *BCL2* overexpression on apoptotic signalling [11, 18] (Fig. 1A). A gene dose-dependent delay of apoptosis measured by PARP cleavage up to the equivalent of 5 extra copies of *BCL2* was seen in these simulations, beyond which the cells survive the full 140 h of the simulation (Fig. 1B). We repeated these simulations in a population of 1000 molecularly heterogeneous individual B cells (Fig. 1C and S1A). In these simulations cell death times retained a log-normal distribution, but 1.5-fold overexpression *of BCL2* increased the mean survival time in individual B cells by 1.3-fold (Fig. 1C). This is consistent with the delayed cell death seen in B cells from *BCL2*-overexpressing mice [21].

*MYC* is a proto-oncogene that codes for a multi-functional transcription factor (cMyc) *with* a prominent role in tumour cell growth, which has previously been modelled as acting on B cell progression within the cell cycle [13, 22]. Simulating cMyc as promoting cell proliferation has been found to recapitulate both single-cell and bulk experimental measurements [10, 11]. We isolated the cell cycle regulatory network from an established model of B cells and simulated the impact of overexpression of *MYC* [11, 18] (Fig. 1D). These simulations predicted that increasing *MYC* expression by the equivalent of 1 extra copy (1.5-fold increase) shortens the cell cycle by just 8 minutes in a 12-h cycle; an effect size much smaller than expected (Fig. 1E). The sensitivity analysis also found that *MYC* expression had a smaller effect on cell cycle time than other genes in the pathway (Fig. S2). Importantly, this result recapitulated experimental tracking of division times in B cells transfected with *MYC* and cellular proliferation measurement in lymphocytes from Eμ-Myc mice [23]. In simulations of increasing levels of *MYC* expression, the small decrease in total cell cycle time was primarily due to a shortening of G1 phase, with a gene dose-dependent effect up to the equivalent of 5-fold higher expression, after which no further effect was seen (Fig. 1E). Upregulating *MYC (x1.5)* in a heterogeneous population of 1000 individual cells confirmed these findings and resulted in simulated cell cycle timings that recapitulated the distribution of cell cycle duration measured by time-lapse microscopy in murine B lymphocytes [24] (Figs. 1F and S1B). In these simulations, increased *MYC* expression was found to have a minor effect in all cells, decreasing total average cell cycle time by just 11.5 minutes in a 12-h cycle (Fig. 1F). The wild-type simulation identified a population of cells (8%) in cell cycle arrest. Concentrations of the cell cycle inhibitor p27, and complexes containing p27, were upregulated in these arrested cells (Fig. S3), which recapitulates experimental measurements of cell cycle arrest [25]. Interestingly, in simulations of 1.5-fold *MYC* overexpression the cell cycle arrest was reversed in over 80% of these cells (Fig. 1G). The number of cells rescued from arrest by *MYC* overexpression was proportional to the level of *MYC* expression (Fig. S4) and these rescued cells became the most rapidly proliferating cells within the cell population (Figs. 1G and S1B). In keeping with these findings, *MYC* is amongst the most differentially expressed genes in quiescence and reverses quiescence in haematopoietic stem cells [25, 26]. Recent data has demonstrated that increasing the abundance of cMyc can enable senescent cancer cells to resume division [27]. Taken together this data shows that computational modelling of the impact of *MYC* and *BCL2* mutations, on the cell cycle and apoptosis respectively, accurately recapitulates multiple experimental measurements.

### Multi-scale modelling predicts that mutations that converge on apoptosis and the cell cycle confer poor prognosis in blood cancer patients

**Fig. 2:**
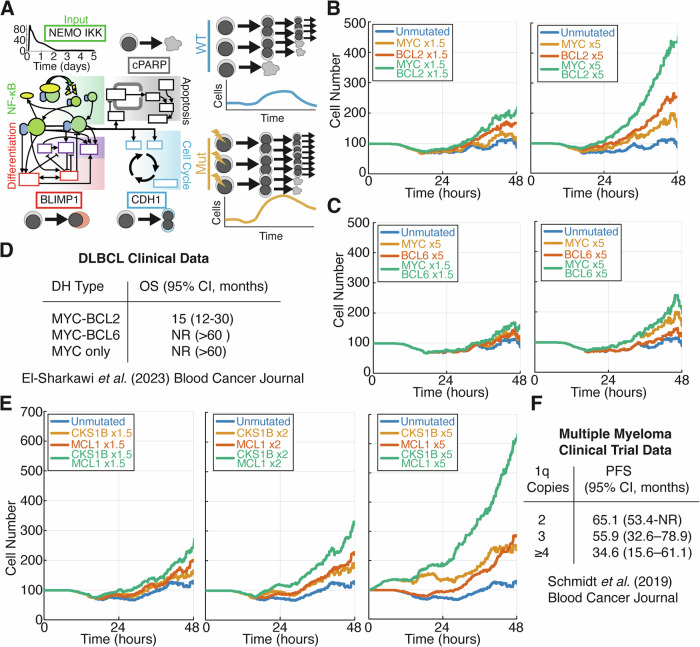
Multi-scale modelling of mutations in subsets of DLBCL and Multiple Myeloma recapitulates clinical trial data. **A** (**left**) Simplified schematic model divided into component signalling pathways featuring NF-κB signalling, apoptosis, the cell cycle and differentiation (adapted from ref. 11), full details provided in Supplementary Material. **A** (**right**) Schematic representation of the progression of cell lineages over time during multiscale simulations. The example depicts a mutation that allows cells to continue to proliferate when they would otherwise die. **B**, **C** Simulated cell population size (cell count) over time for wild-type (blue), individual mutations (orange and red) and double hits where both mutations are combined (green). **D** Overall survival (OS) data for groups of patients with *MYC* and *BCL2*/*BCL6* mutations. **E** Cell population size (cell count) over time for simulations of gain1q multiple myeloma. *CKS1B* and *MCL1* are located on chromosome 1q and therefore amplifications in this chromosome increase the abundance of both of these genes. **F** Progression-free survival (PFS) data for groups of patients with upregulation of *CKS1B* and *MCL1* due to 2, 3 or 4 or more copies of chromosome 1q. NR not reached.

To establish whether computational simulations can accurately predict patient outcomes, we initially simulated the well-characterised subgroup of lymphoma patients with mutations affecting *MYC* and *BCL2* (DH lymphoma). An agent-based multiscale model, previously used to simulate immune responses, was used here [10, 11, 13]. In this framework NF-κB-activation through NEMO:IKK provided the input, resulting in the induction of NF-κB target genes (e.g. *CYCD*, *MYC*, *IRF4*, *BCL2*). The impact of these target genes on the molecular networks controlling cell division and death could trigger the cell cycle to complete mitosis (resulting in daughter cells being added) or the cell to undergo apoptosis (resulting in the cell being removed, Figs. 2A and S5, see methods). Simulations with upregulated (equivalent to 1 extra copy, 1.5-fold increased expression) *MYC* and *BCL2*, resulted in cell number increases due to increased cell division and survival respectively. When both genes were upregulated (1.5-fold expression) cell counts increased in an additive manner (Fig. 2B, left). However, a 4–6 fold increase in expression of *BCL2* and *MYC* is commonly observed in these genes when they are mutated in lymphoma patient samples [28]. Under these conditions (5-fold increased expression), the simulations resulted in an exponential increase in cell numbers (Fig. 2B, right). The classification of DH lymphoma includes mutations in *MYC* and *BCL2* or *BCL6*, but performing the same simulation on the *MYC* + *BCL6* DH lymphoma resulted in a substantially smaller effect compared to the *MYC* + *BCL2* DH (Fig. 2C). Using cell numbers as a proxy for disease prognosis resulted in predictions that *MYC* + *BCL2* DH lymphoma would have worse prognosis than *MYC* + *BCL6* DH lymphoma (Fig. 2B, C). This was confirmed in a multicentre retrospective data collection study [29], which showed that *MYC* + *BCL6* DH lymphoma does not represent high-risk lymphoma, while *MYC* + *BCL2* DH lymphoma has particularly poor prognosis (Fig. 2D).

To determine whether computational predictions could recapitulate disease prognosis in other high-risk haematological malignancies we simulated gain 1q MM (Fig. 2E). Gain 1q is a commonly occurring cytogenetic event in MM associated with therapeutic failure and inferior prognosis [30]. Genes encoding the Bcl2-family protein Mcl1 (*MCL1*) and cell cycle regulator Cks1b (*CKS1B*) both reside on chromosome 1q21 and have been implicated in the pathogenesis of gain1q MM [31]. Simulating the upregulation of each gene individually and in combination revealed a dose-dependent increase in cell numbers as the number of copies of genes on 1q increased (Fig. 2E). Retrospective analysis of clinical data found a dose-dependent worsening prognosis with increasing copies of chromosome 1q [19] (Fig. 2F). Strikingly, with a 5-fold increase in expression of *MCL1* and *CKS1B* the cell population continues to increase beyond the presence of any input signal (IKK activity returns to basal by 72 h, Fig. S6). These simulations indicate that amplification of the chromosomal region containing *MCL1* and *CKS1B* may result in constitutive signalling and microenvironmental independence, which is circumstantially supported by the observation that 1q21 is amplified in 91% of MM cell lines [32].

We noted that within the models we were using, *MYC* and *BCL2* in DH lymphoma impact the cell cycle and apoptotic regulatory networks respectively, while *BCL6* is situated within molecular networks controlling terminal B cell differentiation (Fig. S5). In the context of gain1q MM, *CKS1B* and *MCL1* overexpression also results in the perturbation of the cell cycle and apoptosis respectively. Consistent with DH lymphoma, we found that (in MM) when mutations simultaneously impact the cell cycle and apoptosis they combined deleteriously in simulations and conferred poor prognosis in clinical data [19, 29].

### A computational pipeline enables simulations of heterogeneous patients from WES data by mapping mutations to simulation parameters

**Fig. 3:**
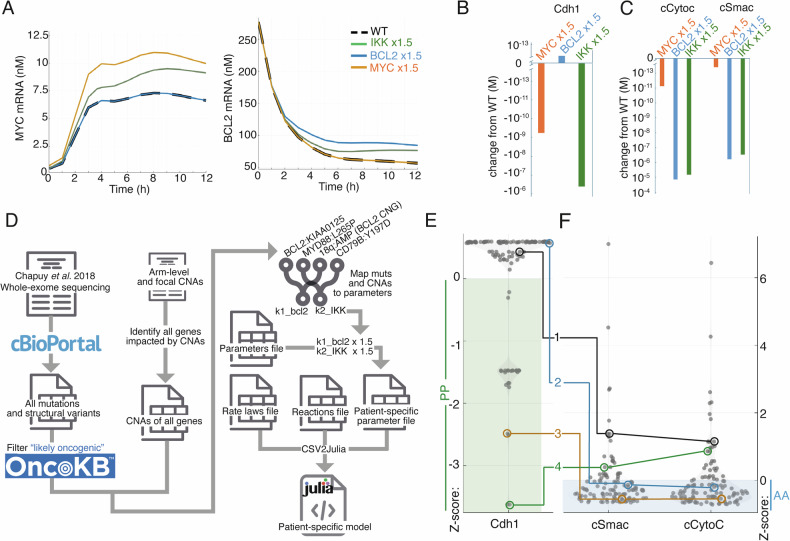
Multiple mutations can create anti-apoptotic and pro-proliferative signalling. **A** Abundance of *MYC* mRNA (left) and *BCL2* (right) mRNA in simulations of wild-type (WT) (dash), an IKK-activating mutation (green), a *BCL2*-activating mutation (blue) and a *MYC*-activating mutation (orange) over time. Note that *BCL2* mRNA is elevated at *t* = 0 as the model transitions from steady state phase (with enforced survival signal) to the dynamic phase (with dynamically-determined survival signal). **B** Changes in the abundance of Cdh1 protein at 6 h in the simulations from (**A**). Each simulated concentration is subtracted from the WT simulation and plotted on a log scale as either an increase or decrease in abundance. Note that both *MYC* and IKK activating mutations decrease Cdh1 indicating a more rapid transition from G1 to S phase. **C** Changes in the abundance of cCytoc (cytoplasmic cytochrome c, left) protein, and cSmac (cytoplasmic second mitochondria-derived activator of caspase, right) at 6 h in the simulations from (**A**). Each simulated concentration is subtracted from the WT simulation and plotted on a log scale as either an increase or decrease. Note that both *BCL2* and IKK activating mutations decrease both cSmac and cCytoc indicating reduced apoptotic signalling. **D** Pipeline to incorporate mutational events from genetic sequencing to create patient-specific models. Example mutational mappings are shown, including the model parameters they modify. The full mapping is provided on in the Github repository (https://github.com/SiFTW/norrisEtAl/blob/main/muts2Params.csv). **E** Violin plot showing the concentration of Cdh1 in individual patient simulations created as shown in (**D**). Each abundance is standardised and displayed as a z-score. The region with below mean abundance of Cdh1 is highlighted in green and labelled as PP (pro-proliferative), with example patients highlighted that are within and outside this region. **F** Concentration of cSmac (left) and cCytoC (right) in individual patient simulations created as shown in (**D**). Each abundance is standardised and displayed as a z-score. The region with below mean abundance of each protein is highlighted in blue and labelled as PP (pro-proliferative), with example patients highlighted that are within and outside this region. Note that patient 1 (LS3593) is neither AA or PP, 2 (RICOVER_977) is only AA, 3 (RICOVER_126) is AAPP, and 4 (LS2305) is PP only.

Having demonstrated that the model can accurately predict known poor prognosis mutation combinations, we next sought to establish whether other co-occurring deleterious mutations that impact both the cell cycle and apoptosis could be identified through computational modelling. Comparing simulations of activating mutations in *BCL2* and *MYC* to simulations of an upstream NEMO:IKK/NF-κB-activating mutation demonstrated that NF-κB-activating mutations could cause an increase in both *BCL2* and *MYC* mRNA similar to that seen in DH lymphomas (Fig. 3A). Upregulation of IKK and *MYC* results in downregulation of the cell cycle regulator Cdh1 at 6 h, 9 h and 12 h (Figs. 1E, 3B and S7), indicating more rapid progression through the G1 phase of the cell cycle, while activating mutations in *BCL2* decrease the release of the apoptosis-inducing proteins cytochrome c and Smac to the cytoplasm (Figs. 3C and S7).

To employ the model to identify additional combinations of mutations that could result in simultaneous anti-apoptotic and pro-proliferative molecular signalling, we sought to leverage a WES dataset that capture the mutational heterogeneity in individual DLBCL patients [7]. We developed a pipeline to map both individual gene-level mutations and chromosomal arm-level copy number alterations to computational parameters in the multi-scale model (Fig. 3D, see Materials and Methods). This process created 113 patient-specific models, with parameters modified based on gene alterations described from analysis of WES data (Fig. 3D), while all other parameters remained the same [33]. Calculating the mean of Cdh1, cytoplasmic Smac (cSmac) and cytoplasmic CytoC (cCytoC) values across 100 cells enabled identification of a subset of patients with below average Cdh1 (pro-proliferative: PP, Fig. 3E example patients 3 and 4), and/or below average cSmac and cCytoC (anti-apoptotic: AA, Fig. 3F example patients 2 and 3). Patients with both anti-apoptotic and pro-proliferative signalling (AAPP) could be identified (Fig. 3E, F example patient 3), along with patients with neither AA or PP signalling states (Fig. 3E, F example patient 1).

### Personalised patient simulations identify an anti-apoptotic and pro-proliferative subgroup of patients with poor prognosis

**Fig. 4:**
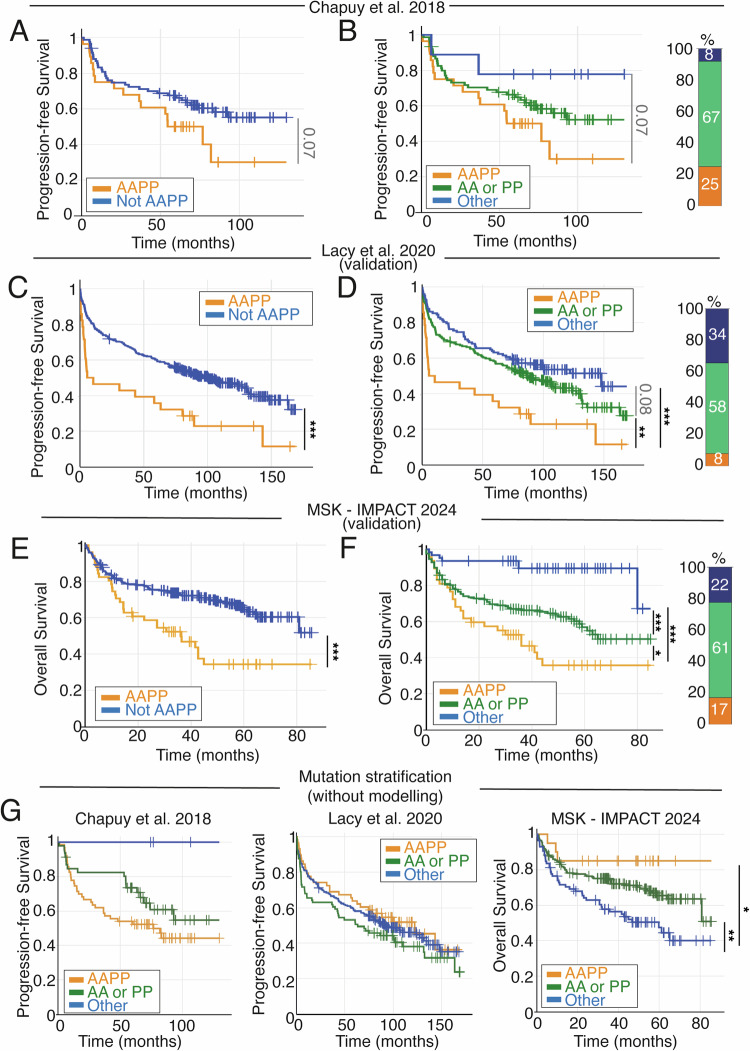
Patient-specific modelling and stratification by pro-proliferation and anti-apoptotic species predicts prognosis of DLBCL patients. Kaplan-Meier (KM) plots were generated using modelling of patient data derived from Chapuy et al. (**A**, **B**), and then validated using Lacy et al. (**C**, **D**), and the MSK IMPACT cohort (E-F). **A** KM plot comparing progression-free survival (PFS) for DLBCL patients classified as simultaneously anti-apoptotic (AA) and pro-proliferative (PP)(AAPP) or not (Other), using personalised simulations. **B** KM plot comparing PFS for DLBCL patients classified as simultaneously anti-apoptotic and pro-proliferative (AAPP, prange), only one of AA or PP (green), or neither (Other, blue), using personalised simulations. The proportion of patients in each group is shown on the right. KM plots generated the same way as (**A**, **B**) using patients from ref. [8], stratified using AAPP alone (**C**), AA and/or PP (**D**). KM plots generated the same way as (**A**–**C**) using patients from the MSK IMPACT Heme cohort (2024), stratified using AAPP alone (**E**), AA and/or PP (**F**). **G** KM plots, generated without modelling in the indicated cohorts, comparing PFS/OS for patients with at least one mutation mapping to each of the cell cycle and apoptotic networks (AAPP, orange), with at least one mutation mapping to either the AA or PP network but not both (AA or PP, green), and patients who don’t have a mutation that maps to either AA or PP signalling networks (Other, blue). Significance values from log rank test indicated as follows: * *P* ≤ 0.05, ** *P* ≤ 0.01, *** *P* ≤ 0.001.

Analysing pro-proliferative (PP) and anti-apoptotic (AA) factors over time for individual patient simulations revealed highly heterogeneous expression between patients, with most dynamic changes occurring in the first 6 h (Fig. 3A). Values at 6 h were chosen to stratify patients as this was the earliest time point at which most transient dynamics had passed but cells had not been removed from the simulation due to cell division or cell death. Furthermore, the abundance of *BCL2* and *MYC* mRNA at later time points correlated strongly with those at 6 h (*R*^2^ > 0.97 between 6 h, 9 h and 12 h time-points for both pro-proliferative and anti-apoptotic factors, Fig. S8). Stratification of patients based on AA and PP signalling in model simulations identified a group of patients (25% of the Chapuy cohort) for which both high anti-apoptotic and pro-proliferative (AAPP) signalling was present. Patients with AAPP signalling (Fig. 4A), using computational simulations, had worse prognosis than other DLCBL patients in this cohort. Reflecting trends seen in earlier simulations of DH DLCBL and gain1q MM (Fig. 2), patients with simultaneous AA and PP signalling had worse prognosis (25% of patients, median PFS 55 months), than patients with either AA or PP signalling (67% of patients, median PFS not reached, 52% PFS at 120 months), while patients with neither AA nor PP signalling (‘other’) had good prognosis (8% of patients; median PFS not reached, 78% PFS at 120 months, (Fig. 4B)).

We sought to validate these findings using data from larger independent cohorts. We therefore repeated this analysis in two additional cohorts: Lacy et al. [8] and the DLBCL subset of the MSK-IMPACT Heme cohort (2024), generating patient-specific models for 354 and 276 patients respectively. Of note, both these studies used targeted sequencing platforms rather than WES and therefore this analysis would validate whether this approach is applicable to panel-based sequencing. Due to the size of these validation datasets, we reduced the size of simulations from averaging 100 single cells to simulating one single-cell per patient. Survival analysis showed consistent significant differences between patients classified as AAPP, compared to all other patients across both validation cohorts (Fig. 4C, E). Furthermore, patients predicted to have concurrent anti-apoptotic and pro-proliferative signals had poor prognosis across all cohorts, while patients bearing either AA or PP signatures had an intermediate prognosis and the best prognosis was seen in patients with neither model-derived signature (Fig. 4B, D, F).

To confirm that mechanistic modelling was truly identifying emergent signalling properties rather than simply counting the presence of mutations that impact apoptotic and proliferative signalling networks, we stratified patients by the presence or absence of mutations that mapped to the anti-apoptotic, or pro-proliferative signalling networks (Fig. 4G). Stratifying patients by simply using the presence or absence of mutations without simulation failed to consistently predict prognosis across our discovery and validation cohorts (Fig. 4G). Indeed, patients with simultaneous mutations impacting apoptotic and proliferative signalling networks had poor prognosis in our validation cohort, no prognostic difference in the Lacy et al. cohort and significantly better prognosis in the MSK cohort (Fig. 4G). Further stratifying patients to identify those with multiple mutations impacting these networks did not improve these predictions (Fig S12). This demonstrates that simulating mutations within the context of signalling pathways can differentiate between co-occurring mutations that do or do not combine deleteriously to confer poor prognosis, with consistent results across multiple cohorts and sequencing modalities.

The hazard ratio (HR) for AAPP patients was 2.58 (95% confidence interval: 1.57–4.220, *p* < 0.001) in the Lacy cohort and 7.07 (3.73–13.41) in the MSK Cohort, when compared to patients not assigned AAPP (Fig. S9 and S10). Comparing AAPP stratification to other prognostic predictors in the MSK cohort model-based stratification out-performed stratifying patients based on whether they were previously treated (HR: 2.1, 95% CI: 1.4–3.1), or by International Classification of Diseases for Oncology staging (HR: 1.3, 95% CI: 0.9–1.9, Fig S9). In the Lacy cohort AAPP patients had worse prognosis than all but the highest IPI category (Fig S10). Importantly, AAPP patients identified by modelling could not be identified from mutational burden alone without simulation and did not differ in their age at diagnosis, IPI score, CNS involvement, mutation count, number of driver mutations, number of copy number alterations, ploidy, or any other clinical parameter available (*q* = 0.1–1.0 for all clinical metrics) compared to the rest of the cohort (Fig. S11).

### Personalised computational simulations are independent of, and can be combined with, IPI, stage and genetic clustering, to improve prognostic power

**Fig. 5:**
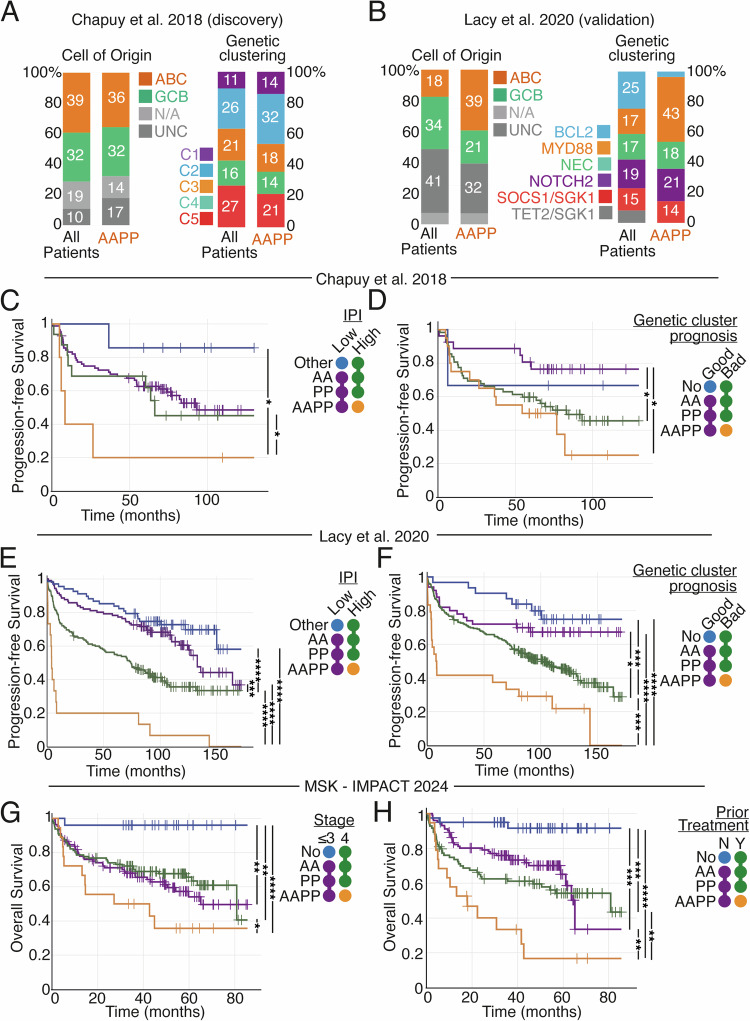
Computational modelling identifies poor prognosis patients in all cell-of-origin and genetic groupings and can be combined with multiple establish prognostic metrics to reliably stratify patients. **A** Grouped bar plot showing the percentage of AAPP and non-AAPP patients in each cell-of-origin (left) genetic cluster from Chapuy et al. [7] (right). C1-5 = cluster 1 to 5 as assigned in the original publicaiton. **B** Grouped bar plot showing the percentage of AAPP and non-AAPP patients in each cell-of-origin (left) genetic cluster from Lacy et al. 2020 [8] (right). Cluster names maintained from Lacy et al. Kaplan-Meier analysis of progression-free survival (PFS) in patients from Chapuy et al. (**C**) and Lacy et al (**E**) stratified into: low IPI and neither AA or PP signalling (blue), low IPI with one or more AA/PP signalling states (purple), high IPI without AAPP signalling (green), high IPI with simultaneous AAPP signalling (orange). Kaplan-Meier analysis of PFS in patients from Chapuy et al. (**D**) and Lacy et al. (**F**) stratified into: good-prognosis genetic cluster and neither AA or PP signalling (blue), good-prognosis genetic cluster with one or more AA/PP signalling states (purple), poor-prognosis genetic cluster without AAPP signalling (green), poor-prognosis genetic cluster with simultaneous AAPP signalling (orange). **G** Kaplan-Meier analysis of overall survival in DLBCL patients from the Memorial Sloan Kettering (MSK) IMPACT - Heme cohort stratified into: stage ≤ 3 with neither AA or PP signalling (blue), stage ≤ 3 with one or more AA/PP signalling states (purple), stage 4 without AAPP signalling (green), stage 4 with simultaneous AAPP signalling (orange). **H** Kaplan-Meier analysis of overall survival in DLBCL patients from the MSK IMPACT - Heme cohort stratified into: no prior treatment with neither AA or PP signalling (blue), no prior treatment with one or more AA/PP signalling states (purple), treatment prior to arrival at MSK without AAPP signalling (green), treatment prior to arrival at MSK with simultaneous AAPP signalling (orange). ABC Activated B Cell, GCB Germinal Centre B cell, N/A no data available, UNC unclassified, NEC Not Elsewhere Classified, IPI International Prognostic Index, Stage = International Classification of Diseases for Oncology staging. Poor prognosis clusters in Chapuy et al: 2, 3 and 5. Poor prognosis clusters in Lacy et al: BCL2, MYD88, NEC, NOTCH2. Low IPI: 1–3 Chapuy et al. and ‘Low’ to ‘Low/Intermediate’ Lacy et al. Significance values from log rank test indicated as follows: * *P* ≤ 0.05, ** *P* ≤ 0.01, *** *P* ≤ 0.001, **** *P* ≤ 0.0001.

AAPP patients were identified in all genomic clusters and cell-of-origin assignments in the discovery cohort and all but one genomic cluster in the Lacy et al. validation cohort (Fig. 5A, B). We next tested whether model-based patient stratification could be combined with existing prognostically predictive clinical and genetic measurements. For each cohort we assigned patients to one of 4 categories (Fig. 5C–H), these were: (1) No AA or PP signalling states combined with a good prognostic metric (blue), (2) Good prognostic metric but the presence of one or more AA/PP signalling states (purple), (3) Poor prognostic metric but without the combined AAPP signalling state (green), (4) Poor prognostic marker and AAPP signalling (orange). We tested with multiple prognostically informative metrics, in all modelled datasets: International Prognostic Index (IPI, Fig. 5C, E), genetic clustering (Fig. 5D, F), International Classification of Diseases for Oncology stage (Fig. 5G), and whether patients were treatment naïve or not (Fig. 5H). Patients with combined high IPI and AAPP signalling had dismal prognosis (median PFS 8.4 months in Chapuy et al. (Fig. 5C); 4 months in Lacy et al. (Fig. 5E)), while patients without high IPI or any AAPP signalling had favourable prognosis (median PFS not reaching, 120-month PFS 86% in Chapuy et al. (Fig. 5C); 73% in Lacy et al. (Fig. 5E)). Patients with combined poor prognostic clusters and AAPP signalling had the worst prognosis in both (median PFS 54 months in Chapuy et al. (Fig. 5C); 7.8 months in Lacy et al. (Fig. 5E)), while patients in good prognosis genetic clusters without any AAPP signalling had positive outcomes (medial PFS not reached, 120-month PFS 76% in Chapuy et al. (Fig. 5C); 75% in Lacy et al. (Fig. 5E)). Stratification was robust to the inclusion or exclusion of patients that did not contain a single mutation that mapped to a model parameter (Fig. S13), and to the techniques (Akaike information criteria or integrated completed likelihood) used to identify the genomic clusters (Fig. S14).

Neither IPI nor genetic clustering had been performed in the MSK IMPACT cohort, however data on whether patients had received prior treatment and their disease staging were available. Patients with stage 4 disease and AAPP signalling had poor prognosis (median overall survival (OS) 27 months, (Fig. 5G)), while patients with stage 3 or lower and no AAPP signalling had strikingly good prognosis (median PFS not reached; 96% 60-month OS, (Fig. 5G)). Similarly, patients whose disease was not treatment naïve with AAPP signalling had dismal prognosis (median OS 17.9 months), while patients with treatment naïve disease and no AAPP signalling had remarkably good prognosis (median OS not reached; 92% 60-month PFS). Comparing each of these prognostic stratifications with (Fig. 5C–H) and without (Fig. S15) personalised computational models demonstrates that modelling identifies novel groups of patients with strikingly good and poor prognosis that are not identified without computational modelling.

## Discussion

Predicting which blood cancer patients will respond well to treatment is key to empowering both patient and clinician in clinical decision-making. Reliable predictive tools could also inform clinical trains designed to advance the standard of care in heterogeneous blood cancers. In this study, we found that leveraged genomic sequencing data from DLBCL patients to create personalised simulations that quantified the impact of mutations on anti-apoptotic and pro-proliferative signalling. This enabled reliable prognostic prediction across multiple datasets despite widespread heterogeneity in mutational burden. Molecular clustering of DLBCL has previously identified clusters of patients that differ in the signalling pathways most impacted by mutations [7–9]. However, a single mutation may impact multiple signalling networks and multiple mutations may overcome or exacerbate each other. For example, gain of 19q increases *BAX* (pro-apoptotic), *CCNE1* (pro-proliferative) and *NFKBIB* (pro-apoptotic and anti-proliferative). Computational modelling enabled the net cellular consequence of mutational landscapes of individual patients to be determined. In particular, our models identified patients with co-occurring mutations that are simultaneously pro-proliferative and anti-apoptotic, which could not be determined from mutational clustering or the presence or absence of specific mutations. Importantly, this modelling approach identified patients with dismal, intermediate and good prognosis across multiple datasets, using either WES or targeted sequencing panels [34]. While statistical approaches such as artificial intelligence tend to perform worse on validation data than training data, we found that results from simulation-enabled stratification became more significant in larger validation datasets [34]. We believe this highlights the importance of encoding molecular network knowledge to contextualise mutational information [12, 35]. We also demonstrate that results from simulations can be combined with genetic clustering and a variety of clinically used prognostic tools to identify novel subgroups of patients with both dismal and favourable prognosis. As such, model-enhanced stratification could be a valuable tool to inform risk-stratified trials in the future.

There is compelling evidence supporting the adoption of genetic sequencing at diagnosis of DLBCL to determine prognosis [36, 37]. All mutations and CNAs modelled in this study affected recurrently mutated genes, which are profiled by targeted sequencing panels. As such, the data required for modelling could be generated at low cost and from fixed diagnostic samples and potentially even a plasma sample [38]. Furthermore, the recent development of alternatives to R-CHOP is likely to motivate utilisation of genomic sequencing data to rationally assign therapies. There is emerging evidence supporting the addition of Polatuzumab vedotin for high-risk DLCBL, the addition of Bortezomib in ABC-DLBCL, or the use of dose-adjusted EPOCH plus Rituximab for patients expressing high levels of *MYC* and *BCL2* [39–41]. Factoring in the decreasing costs of sequencing, it seems likely that molecular profiling will become standard diagnostic practice in DLBCL, with the aim of precisely identifying patients that would benefit from an alternative to R-CHOP. Here we show that computational modelling can improve the ability to identify such patients without requiring additional data.

Substantial computational work was performed in this study, much of which would be infeasible to perform in regular clinical practice. Therefore, when simulating the larger validation datasets, we tested whether a single simulation of a single-cell could achieve the same significant prognostic power. These simulations take a few minutes to complete for each patient on consumer CPUs. Such analysis could be performed on computers available in clinical institutions using the code available with this study, and free Julia software. In future development of an online portal where sequencing data can be uploaded and analysed remotely may further expedite clinical use of personalised models.

More work is required if modelling is to become a widely used tool for personalised medicine approaches. Here we chose not to create new models, but rather to test the utility of established computational models of B cells that have not previously been applied to lymphoma [10, 11]. We performed no parameter fitting, as these parameters have been accumulated and validated across multiple cellular contexts and cell types [10, 12, 13, 35]. We assume that parameters, other than those affected by mutations, remain consistent between healthy B cells and cancerous B cells. We also assumed that all mutations within patients have the same effect size, equivalent to one extra copy or loss of copy of each gene. The reality will be more complex and we expect that identifying and quantifying the impact of each mutation on each gene would improve the utility of the model. Increasing the scope of the simulations, such that more recurrent mutations can be directly assigned to model parameters is also likely to improve the utility of this approach. However, the current model is clearly able to identify a population of very poor prognostic patients that would not have been identified using mutational clustering and cell of origin (COO) alone.

Gene expression-based classifiers have also been used to identify high-risk DLBCL cases such as molecular high grade (MHG) DLBCL and dark zone signature (DZsig) DLBCL [42, 43]. Of note, both of DZsig and MHG patients are almost exclusively classified by COO classifiers as GC-DLBCL. Model-based classification does not enrich for GC-DLBCL when identifying high risk patients. It also does not enrich for patients with other poor-prognosis clinical characteristics such as IPI (enriched in MHG), stage (enriched in MHG and DZSig), or lactate dehyrodgenase (enriched in DZsig) indicating that simulations enable identification of high-risk patients that could not otherwise be identified.

The B cell receptor and toll-like receptor signalling pathways, although not explicitly modelled here, converge on NEMO:IKK and are the target of new therapeutic advances and recurrent mutations. Similarly, epigenetic regulators such as EZH2 are recurrently mutated but not explicitly modelled. Further work is also required to build on this model and simulate how treatment may perturb biological networks harbouring mutations. Such approaches may provide mechanistic insight into the development of relapsed DLBCL and provide insight into second-line treatments. Simulating the effect of targeted inhibitors in the patient-specific simulations presented here may provide insights into which patients will respond to alternatives to R-CHOP, however, limited clinical data is available to validate such predictions.

Performing large-scale computational simulations can be computationally challenging and require substantial computational resources given the size and complexity of the molecular networks simulated here (194 equations, and 563 parameters). Previous work has applied logical modelling (molecular components are discretised into high/medium/low) to B cell lymphoma to overcome this challenge [44]. Here, we found a continuous modelling approach was able to identify many gene dose-dependent effects that could not be identified with logical modelling, including how chromosomal gain and amplification confer distinct prognoses. Despite the requirement for substantial computational calculation, we found that simulating just 6 hours of a single B cell per patient (simulating 100 cells required ~30 minutes of real-word time on a 2.1 GHz CPU) enabled us to stratify patients. The ability to perform short simulations is enabled by experimental results that demonstrate that B cell molecular network states are rapidly established and reliably inherited across multiple generations of cell division [10]. It is likely that clinical implementations of computational modelling could provide additional insight based on simulations without specialised hardware and without delaying treatment.

Beyond DLBCL and MM, the modelling methodology described here may have utility in other tumour types when mutational data and comprehensive, experimentally validated, simulations are available. Many of the recurrent mutational events in DLBCL and MM are common cancer-associated mutations. *MYC* deregulation is involved in the development of a wide variety of cancers [45], *BCL2* is dysregulated in many malignancies including breast cancer and gastric carcinoma [46, 47], and NF-κB is implicated in numerous cancers [48]. A mathematical model, simulating 17 cancer types, estimated that the number of carcinogenic mutations (hits) varies from two to eight depending on the cancer type [49]. With the increasing availability of cancer genome data and the evolution of computational methods to identify the mutational burden of cancer patients, the approach described here is rapidly becoming feasible in numerous cancers. We expect these approaches to be most impactful in mutationally heterogeneous cancers, such as breast cancer, where heterogeneity challenges early diagnosis, treatment selection and prognosis prediction [50].

The ultimate goal of computational systems biology approaches such as the one presented here is to enable personalised medicine by using models to identify the right drugs for the right patients. Modern sequencing techniques provide an abundance of data, but we have yet to fully utilise it. Computational models with the power to translate patient-specific mutation data into personalised prognostic and treatment predictions may empower the exploitation of these data to enable truly personalised medicine approaches.

### Supplementary information

Supplementary Material

## Data Availability

The datasets and computer code produced in this study are available in the following databases: Modelling computer scripts: GitHub (https://github.com/SiFTW/norrisEtAl).
